# Combined Statin and Glucocorticoid Therapy for the Safer Treatment of Preterm Birth

**DOI:** 10.1161/HYPERTENSIONAHA.122.19647

**Published:** 2023-02-01

**Authors:** Andrew D. Kane, Emilio A. Herrera, Youguo Niu, Emily J. Camm, Beth J. Allison, Deodata Tijsseling, Ciara Lusby, Jan B. Derks, Kirsty L. Brain, Inge M. Bronckers, Christine M. Cross, Lindsey Berends, Dino A. Giussani

**Affiliations:** Department of Physiology, Development and Neuroscience, University of Cambridge, United Kingdom (A.D.K., E.A.H., Y.N., E.J.C., B.J.A., C.L., K.L.B., C.M.C., D.A.G.).; Laboratory of Vascular Function & Reactivity, Pathophysiology Program, ICBM, Faculty of Medicine, Universidad de Chile, Santiago, Chile (E.A.H.).; The Ritchie Centre, Hudson Institute of Medical Research, Clayton, Victoria, Australia (E.J.C., B.J.A.).; Perinatal Center, University Medical Center, Utrecht, the Netherlands (D.T., J.B.D.).; Institute of Metabolic Science, University of Cambridge Metabolic Research Laboratories, Addenbrooke’s Hospital, Cambridge, United Kingdom (L.B.).; Department of Obstetrics and Gynecology, Radboud University Nijmegen Medical Centre, the Netherlands (I.M.B.).; The Cambridge BHF Centre for Research Excellence, Cambridge, United Kingdom (Y.N., D.A.G.).; The Cambridge Strategic Research Initiative in Reproduction, Cambridge, United Kingdom (Y.N., D.A.G.).

**Keywords:** cardiovascular diseases, glucocorticoids, hypertension, infant, newborn, premature birth

## Abstract

Prematurity is strongly associated with poor respiratory function in the neonate. Rescue therapies include treatment with glucocorticoids due to their anti-inflammatory and maturational effects on the developing lung. However, glucocorticoid treatment in the infant can increase the risk of long-term cardiovascular complications including hypertension, cardiac, and endothelial dysfunction. Accumulating evidence implicates a molecular link between glucocorticoid excess and depletion of nitric oxide (NO) bioavailability as a mechanism underlying the detrimental effects of postnatal steroids on the heart and circulation. Therefore, combined glucocorticoid and statin therapy, by increasing NO bioavailability, may protect the developing cardiovascular system while maintaining beneficial effects on the lung.

**Methods:**

We investigated combined glucocorticoid and statin therapy using an established rodent model of prematurity and combined experiments of cardiovascular function in vivo, with those in isolated organs as well as measurements at the cellular and molecular levels.

**Results:**

We show that neonatal glucocorticoid treatment increases the risk of later cardiovascular dysfunction in the offspring. Underlying mechanisms include decreased circulating NO bioavailability, sympathetic hyper-reactivity, and NO-dependent endothelial dysfunction. Combined neonatal glucocorticoid and statin therapy protects the developing cardiovascular system by normalizing NO and sympathetic signaling, without affecting pulmonary maturational or anti-inflammatory effects of glucocorticoids.

**Conclusions:**

Therefore, combined glucocorticoid and statin therapy may be safer than glucocorticoids alone for the treatment of preterm birth.

**Novelty and Relevance**

**What Is New?**

- In the treatment of preterm birth, combined statin and glucocorticoid therapy is safer than glucocorticoids alone.

**What Is Relevant?**

- Despite life-saving effects on the lung of glucocorticoid treatment in the infant, evidence suggests off-target effects, which can increase the risk of long-term cardiovascular problems in the offspring including hypertension, cardiac and endothelial dysfunction.

**Clinical and Pathophysiological Implications?**

We show that statin therapy protects against adverse effects of glucocorticoids on the developing cardiovascular system by normalizing nitric oxide bioavailability and sympathetic signaling, without affecting beneficial effects of glucocorticoids on the developing lung. Therefore, combined glucocorticoid and statin therapy may improve current clinical practice in the treatment of preterm birth.

Preterm birth causes respiratory distress in the infant and is strongly associated with bronchopulmonary dysplasia and chronic lung disease, secondary to lung immaturity and inflammation during mechanical ventilation.^1,2^ The clinical use of glucocorticoids to treat preterm babies has become common practice because of their maturational and anti-inflammatory effects on the lung.^1,3–5^ Glucocorticoids improve respiratory function through alveolar maturation and induce the production of surfactant, which reduces respiratory work and increases functional residual capacity by promoting alveolar stability.^6^ It is now well established that antenatal^7^ and postnatal^8^ glucocorticoids reduce the incidence of chronic lung disease and dependence on assisted ventilation in preterm infants.

Despite well-documented life-saving effects of perinatal glucocorticoid therapy on lung function, there is growing concern because of lasting adverse off-target effects reported in humans and in preclinical animal models.^8^ Glucocorticoid treatment stunts growth in human infants^9^ and other animals.^10^ Furthermore, hypertension,^10,11^ impaired endothelial function^12^ and cardiac remodeling and dysfunction are all present.^13–15^ Bal et al^16^ performed histopathological and immunohistochemical studies on hearts of rats killed 4, 8, and 50 weeks after a human clinically relevant course of neonatal dexamethasone treatment and compared the findings with a vehicle-treated group of rats to isolate cardiac effects in the prepubertal and postpubertal periods as well as the equivalent of middle-age. That study showed increased cardiomyocyte hypertrophy and collagen deposition in the Dexamethasone-treated rats of all 3 age groups, being most pronounced in the 50-week-old rats.^16^

Accumulating evidence suggests that one pathway via which glucocorticoids may promote their deleterious effects is through decreased nitric oxide (NO) bioavailability.^17^ Clinically relevant glucocorticoid therapy can promote excess reactive oxygen species generation, quenching NO, and their adverse effects can be ameliorated by treatment with antioxidants.^12,15–18^ Glucocorticoids also impair NO function through decreased eNOS (endothelial nitric oxide synthase) mRNA^19^ or by limiting the availability of tetrahydrobiopterin, a key cofactor promoting NO signaling.^20^ In turn, impaired NO bioavailability can promote sympathetic hyper-reactivity, thereby perpetuating cardiovascular dysfunction.^21–23^ Therefore, maintenance of appropriate NO signaling during clinical glucocorticoid therapy may be key to preserve beneficial effects of glucocorticoids on the developing lung while minimizing adverse side effects on the developing cardiovascular system.

A separate line of research shows that the group of the HMG-CoA (3-hydroxy-3-methylglutaryl coenzyme-A) reductase inhibitors, or statins, have become one of the most widely prescribed drugs for the prevention of cardiovascular disease.^24^ In addition to their lipid-lowering effects, so-called pleiotropic advantageous actions of statins on cardiovascular function have been reported, including decreases in arterial stiffness,^25^ reductions in platelet aggregation,^26^ ameliorating sympathetic hyper-reactivity,^27^ and improvements in endothelial function.^28^ These benefits of statins on the cardiovascular system are accredited to increases in NO bioavailability.^29^ This is because statins inhibit the generation of pyrophosphorylated intermediates of the mevalonate pathway, which act as important cofactors for intracellular GTPases (GTP hydrolase enzymes), such as Ras, Rac, and Rho (Rho family of small G proteins).^30^ Such GTPases impair NO bioavailability through promoting eNOS mRNA degradation^31,32^ and breaking down Akt (protein kinase B), a key activator of NOS.^32^ Therefore, by inhibiting the synthesis of GTPases that themselves inhibit NO biology, statins maintain NO bioavailability.

Therefore, in this study, we have intertwined these two lines of thinking to raise the hypothesis that combined dexamethasone and pravastatin therapy is safer than dexamethasone alone in the treatment of preterm birth. This hypothesis was tested by investigating the effects of a human clinically relevant dosing regimen of neonatal glucocorticoid therapy with and without pravastatin treatment on growth, cardiovascular function, and pulmonary maturation in the weanling rat.

## Methods

The authors declare that all supporting data are available within the article.

### Data Availability

Further information and requests for resources and reagents should be directed to and will be fulfilled by the Lead Contact, D.A. Giussani (dag26@cam.ac.uk). This study did not generate new unique reagents. This study did not generate/analyze any datasets or code.

### Animals

All procedures involving animals were carried out under the Animals (Scientific Procedures) Act 1986 and were approved by the Ethical Review Board of the University of Cambridge. The experimental design was conducted in accordance with the Animal Research: Reporting of In Vivo Experiments guidelines. Time-mated pregnant Wistar rats (Charles River Limited, United Kingdom) were delivered to the University of Cambridge between 10 and 14 days of gestation and were individually housed under standard conditions (21±1 °C, 55% humidity, 12-hour/12-hour light/dark cycle) with free access to food and water. At birth (postnatal day [P] 0), pups from each litter were sexed, weighed, and the litter size culled to 8 (4 males and 4 females chosen at random) to standardize feeding and maternal care, as previously described in detail.^12,15^

### Experimental Design

**Figure 1.**
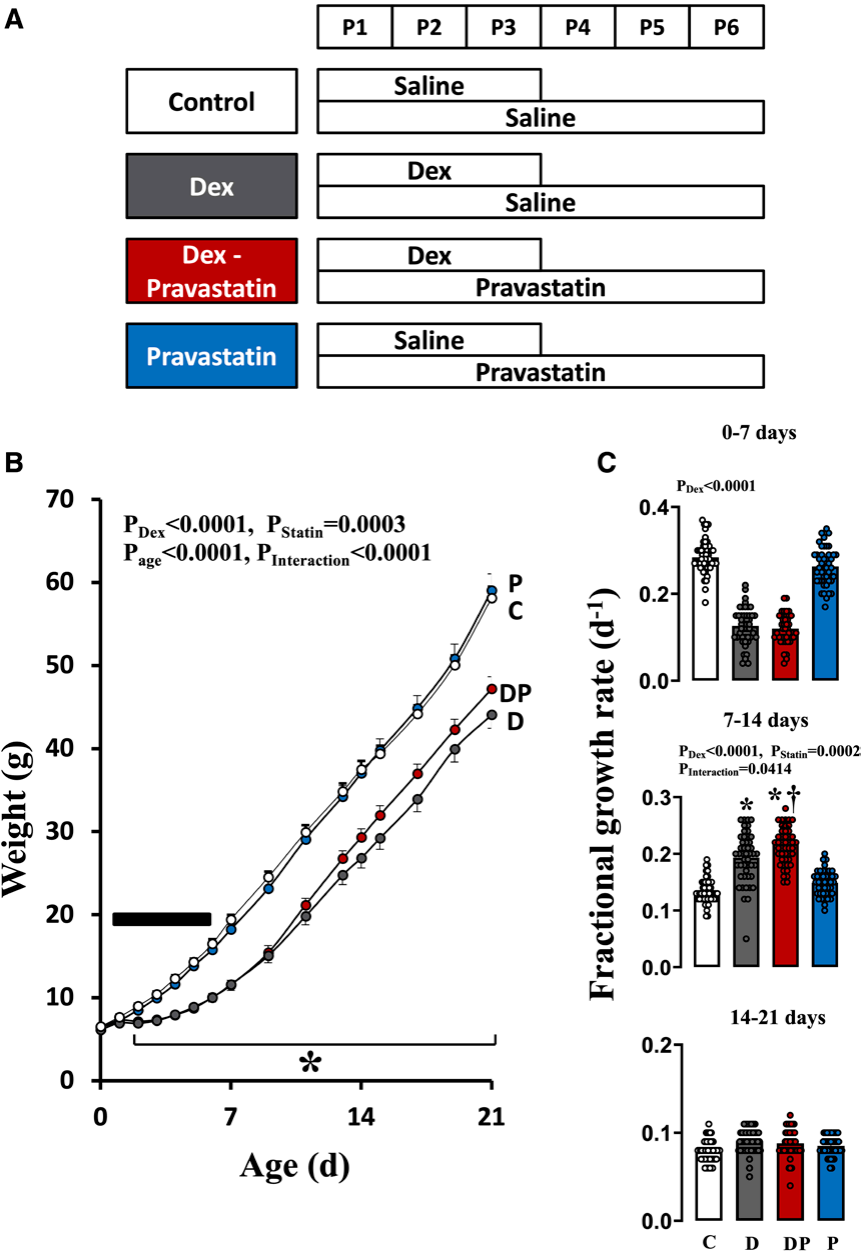
**Experimental protocol and postnatal growth. A**, On postnatal day 1 (P1) to P3 all male pups received either saline (10 µL g^−1^ IP) or a 3-day, tapering course of dexamethasone (Dex; 10 µL g^−1^ IP of solution equating to 0.5, 0.3, and 0.1 mg kg^−1^ dissolved in saline IP). In addition, on postnatal days P1 to P6 all male pups received either saline or pravastatin (10 mg kg^−1^, pravastatin sodium; Sigma-Aldrich, United Kingdom). **B**, Body weight and (**C**) fractional growth rate in pups from control (, C, n=12 litters), Dex-treated group (, D, n=12 litters), Dex and pravastatin–treated group Dex/pravastatin (, DP, n=12 litters) and pravastatin-treated group (, P, n=12 litters) treatment groups. The data represent one male rat per litter per outcome variable. For outcome variables, 1 male rat per litter was used for the in vivo studies, 1 for the isolated organ experiments, 1 was perfusion fixed and 1 was used to harvest tissues that were frozen for molecular studies. Therefore, each experimental group has biometry data from 4 pups per litter. Values are mean±SEM. Black bar signifies the 6-day treatment period. The *P* values for the main factors and interaction in the 2- or 3-way ANOVA are shown. Significant differences (*P*<0.05) for the post hoc Student-Newman-Keuls are *vs control. †D vs DP.

Only male rat pups were studied to control for possible sex differences.^33,34^ On P1 to P3, male pups received either saline (10 µL g^−^^1^ IP) or a 3-day, tapering course of dexamethasone (10 µL g^−^^1^ IP of dexamethasone phosphate disodium salt solution; Sigma-Aldrich, United Kingdom, in 0.9% saline equating to 0.5, 0.3, and 0.1 mg kg^−^^1^ d^−^^1^; Figure 1). In addition, on P1-P6 all male pups received either saline (10 µL g^−^^1^ IP) or pravastatin (10 mg kg^−^^1^ d^−^^1^ in 10 µL g^−^^1^ saline IP Pravastatin sodium; Sigma-Aldrich, United Kingdom). Therefore, the experimental design consisted of 4 treatment groups: control, dexamethasone, dexamethasone+pravastatin, and pravastatin alone. Statin treatment was extended for an extra 3 days after glucocorticoid administration to counteract any possible lag in effects. The volume of each injection was 10 µL g^−^^1^. The dosing regimen of dexamethasone used in this study is proportional to the postnatal 21-day tapering course used in human preterm babies to treat chronic lung disease.^1^ The dose of pravastatin is an intermediate level between human clinical studies and effective doses used in preclinical models.^35^

Pups were individually marked and weighed daily from P0 to P7 and every other day thereafter. Following treatment, pups remained with their mothers until P21. To control for within-litter variation, one male pup per litter was used for any one outcome variable. Therefore, one male was used for in vivo cardiovascular function testing, one male was used for isolated organ experiments (Langendorff preparation and in vitro wire myography), one male was perfusion fixed for histology, and the last male was used for molecular biology studies. A blood sample by cardiac puncture was also taken from this last male to measure circulating agents.

### In Vivo Cardiovascular Function Testing

At P21, 1 male pup per litter was anesthetized with urethane (1.4–1.5 g kg^−^^1^ IP in water for injections; Sigma, United Kingdom). Urethane was chosen to minimize cardio-respiratory depression and permit controlled manipulation of the cardiovascular system under anesthesia in vivo, as previously described.^36–38^ The animal was placed in the supine position on a regulated heating mat, breathing room air spontaneously. Adequate depth of anesthesia was continually assessed by the absence of corneal and limb withdrawal reflexes. When depth of anesthesia was confirmed, under a dissecting microscope, the left femoral artery was exposed via an incision, isolated, and instrumented with a catheter prefilled with heparinized saline (80 IU heparin mL^−^^1^ in 0.9% NaCl). The arterial catheter was connected to a pressure transducer (Argon Division, Maxxim Medical, Athens, TX). Arterial blood pressure was recorded continually using a custom-built Data Acquisition System (Maastricht - Programmable AcQuisition system, M-PAQ and IDEEQ software, Maastricht Instruments, The Netherlands; 1000 Hz sample rate). Heart rate was calculated on-line by the program using the arterial blood pressure pulse as a trigger.

Following a period of stabilization of 60 minutes, basal arterial blood pressure and heart rate were recorded continuously for at least 10 minutes. Heart rate variability was analyzed according to standardized methods.^39–41^ In brief, a 5-minute period of baseline recording was selected and analyzed using the heart rate variability function in Labchart 7 (ADI instruments). Normal to normal (NN) interval was calculated from the arterial blood pressure trace. In the time domain, the square root of the mean of the sum of the squares of difference between adjacent NN intervals, an established parasympathetic index, was calculated.^39–41^ In addition, the data were fast Fourier transformed to determine frequency-specific components. The low-frequency component, an established sympathetic dominant domain, was set between 0.1 and 1.0 Hz. The high-frequency component, an established parasympathetic dominant domain, was set domain between 1.0 and 3.5 Hz. The ratio of the low-frequency/high-frequency power was calculated as an index of autonomic balance, as previously described.^41^

### Isolated Organ Experiments

#### Isolated Langendorff Heart Preparation

At P21, rats were euthanized by CO_2_ inhalation and posterior cervical dislocation, and the heart was rapidly excised and placed in ice-cold Krebs-Henseleit bicarbonate buffer. It was then cannulated via the aorta (<2 minutes from excision) and retrogradely perfused through the coronary arteries at 65 mm Hg with Krebs-Henseleit bicarbonate buffer solution (120 mmol L^−1^ NaCl; 4.7 mmol L^−1^ KCl; 1.2 mmol L^−1^ MgSO_2_.7H_2_O; 1.2 mmol L^−1^ KH_2_PO_4_, 25 mmol L^−1^ NaHCO_3_, 10 mmol L^−1^ glucose, and 1.3 mmol L^−1^ CaCl_2_.2H_2_O; bubbled with 95%:5% O_2_:CO_2_ mix and warmed to 37 °C). A small flexible non-elastic balloon was inserted into the left ventricle (LV) through the left atrium. The balloon was filled with saline and attached to a rigid saline-filled catheter connected to a calibrated pressure transducer (Argon Medical Devices, TX). The balloon volume was adjusted to get a recording of LV end-diastolic pressure of ≈5 mmHg.^42,43^ After an initial stabilization period of 15 minutes, basal heart rate, basal left ventricular systolic pressure, and basal left ventricular end-diastolic pressure were recorded. Basal LV developed pressure was calculated as LVSP–LV end-diastolic pressure. The maximum and minimum first derivatives of the left ventricular pressure with respect to time (dP/d*t*_max_ and dP/d*t*_min_) were calculated using an M-PAQ data acquisition system (the Netherlands). Coronary flow rate was calculated by timed collections of perfusate.^42,43^ The heart was then challenged to a 15-minute period of global ischemia by stopping the perfusion, followed by 30 minutes of reperfusion. The chronotropic and inotropic responses were recorded during recovery from ischemia.

#### In Vitro Wire Myography

At P21, the same animal used for the isolated Langendorff preparation was used for the in vitro wire myography studies. The first branch from the femoral artery of the left hind limb (approximate dimensions: internal diameter, 250 μm; external diameter, 440 μm) was dissected under a bifocal dissecting microscope (Brunel Microscopes Ltd) and placed in ice-cold saline solution. The vessel was carefully cleaned of excess connective tissue and cut into a 2 mm ring. Two 40 μm diameter stainless steel wires were then threaded through the lumen of the femoral sections, maintaining the endothelium intact. The wires were then placed between the mounting support jaws of a 4-chamber small-vessel wire myograph (Multi Wire Myograph System 610M; DMT, Aarhus, Denmark) containing warmed oxygenated Kreb buffer (NaCl 118.5 mM, Fisher, KCl 4.75 mM, Sigma, MgSO_4_.7H_2_0 1.2 mM, Sigma, KH_2_PO_4_ 1.2 mM, Sigma, NaHCO_3_ 25.0 mM, Sigma, CaCl_2_ 2.5 mM, Sigma, glucose 11.1 mM, Sigma, United Kingdom, gassed with 95% O_2_/5% CO_2_ mix, 37 °C).

Force data from the myograph were recorded at 4 Hz (Labchart 6.0, Powerlab 8/30; AD Instruments, Chalgrove, United Kingdom), and each vessel was standardized to an optimal working tension of 80 mmHg, as previously described in detail.^12,44,45^ Cumulative concentration-response constrictor curves to phenylephrine (10^−10^–10^−5^ mol L^−^^1^) were determined in half-log increments. Arteries were given 15- to 20-minute resting and equilibration period between doses. The responses to phenylephrine were normalized to the maximal response to 125 mM K^+^ of the same vessel (percentage of potassium maximal contraction [%K_max_]). The relaxant effects of the endothelium-dependent agonist methacholine (10^−10^–10^−6^ mol L^−^^1^) were then determined after precontraction (phenylephrine; 10^−5^ mol L^−^^1^). To determine the partial contributions of endothelial function mediated via NO-dependent and NO-independent mechanisms, additional concentration-response dilator curves to methacholine were also determined after incubation with N(ω)-nitro-L-arginine methyl ester (10^−5^ mol L^−1^). The contribution of NO-dependent mechanisms to the relaxation induced by methacholine was calculated by subtracting the area under the curve for methacholine—the AUC for methacholine+N(ω)-nitro-L-arginine methyl ester. The contribution of NO-independent mechanisms was calculated by the AUC for methacholine+N(ω)-nitro-L-arginine methyl ester.^12,44,46^

### Histology

At P21, another male was anesthetized with sodium pentobarbital (0.1 mL IP Pentoject; Animalcare Ltd, York, United Kingdom). The heart was exposed through the ribs and an incision was made in the right atrium. The animal was then perfusion fixed at a constant pressure of 60 to 80 mm Hg^45^ with PBS to clear the blood from the circulation and then 10% formaldehyde via a needle inserted into the left ventricle. A section of the fixed thoracic aorta, at the level of the cardiac valves, and the fixed heart were stored in formaldehyde for 24 hours, and then in PBS at 4^o^C until processing. Fixed hearts and aortic segments were sectioned at 5 µm using a microtome (Leica RM 2235, Germany). Ten sections per animal were stained with Masson Trichrome. Cardiac sections were chosen at the level of the cardiac valves. Slides were coded to ensure blinded analysis avoiding bias. The area of the wall and lumen were determined using point counting.^15,46,47^ Points falling on either the wall or lumen were counted to calculate areas as:

A(obj) = a(p) × ΣP

where A(obj) is the estimated area, a(p) is the area associated with each point, and ΣP is the sum of points falling on the relevant area, averaged over the number of sections.

The right lung from each animal was sectioned at 5 μm along the longitudinal axis using the Leica microtome. Fifteen sections were collected from each animal, with the starting section being randomly selected. Selected slides were stained using hematoxylin and eosin stain to visualize the general morphology of the lung parenchyma. The lung tissue to airspace ratio was determined using the point grid system. Secondary septal crest density was also established using a point-counting technique and expressed as a percentage of the total lung tissue area. Three fields of view per section were used for these analyses.

### Molecular Biology Experiments and Biometry

At P21, following euthanasia by posterior cervical dislocation, a cardiac blood sample was taken, aliquots were centrifuged, and the supernatant was stored at −80 °C until further analysis (see below). Body weight and crown-rump length were measured to calculate ponderal index (weight/height^3^). Head diameter was also recorded. The heart, liver, lungs, and brain were dissected and weighed. The heart was divided into left ventricle+septum and right ventricle and these compartments were also weighed separately. Tissues were then snap-frozen in liquid N_2_ and stored at −80 °C. The cardiac left ventricle was then processed for molecular indices of oxidative stress: 4-hydroxynonenal and the Hsp70 (heat shock protein 70). Samples of frozen left lung were processed for indices of pulmonary maturation: SP-C (surfactant protein C) and SP-D (surfactant protein D). Protein aliquots (10–20 µg) resolved on 10% to 12% SDS-PAGE gels were used for Western blot analysis. Tissue homogenization to obtain protein lysates and subsequent SDS-PAGE and immunoblotting were performed, as previously.^15,48–50^ Purified antibodies to β-actin or Ponceau S (1:40 000; Sigma-Aldrich, United Kingdom), 4-hydroxynonenal (1:1000; Abcam, Cambridge, United Kingdom), Hsp70 (1:1,000, Stressgen Bioreagents, United Kingdom) SP-C (1:1,000, Abcam; Cambridge, United Kingdom) and SP-D (1:1,000, Abcam; Cambridge, United Kingdom) in 5% milk in 0.1% Tween 20 detergent were added and then incubated at 4 °C overnight. Membranes were washed in 0.1% Tween 20 detergent, incubated for 1 hour in a secondary antibody conjugated to horseradish peroxidase (donkey anti-rabbit IgG or sheep anti-mouse IgG; 1:10 000, GE Healthcare, United Kingdom) and washed in 0.1% Tween 20 detergent. Proteins were visualized (enhanced chemiluminescence, Amersham, United Kingdom), exposed to X-ray film, and films developed (Fuji FPM100A Processor). For the cardiac molecular studies, the density of bands was quantified and the ratio of each protein to the housekeeping gene was calculated for each sample (ImageJ software, National Institutes of Health).

### Circulating Agents

#### Plasma NO Species

Aliquots of plasma from the blood sample taken at P21 by cardiac puncture above were processed for measurement of plasma concentrations of NO_2_^−^ and NO_3_^−^ (NO_x_ [NO species]), as indices of circulating NO bioavailability,^51^ and plasma concentrations of CRP (C-reactive protein), as an inflammatory marker.^52^ Plasma concentrations of NO_2_^−^ and NO_3_^−^ were determined by a commercially available Nitrate/Nitrite Colorimetric Assay Kit (Catalog No. 780001; Cayman Chemical). In brief, total NO_x_ was measured from plasma samples which were ultra-filtrated to reduce the background interference due to any hemoglobin present. Assay buffer, nitrate standards, and 40 μL of plasma samples were then loaded in duplicate into a 96-well microplate with an enzyme cofactor and nitrate reductase before a 60-minute incubation at room temperature. Following further addition of the Griess reagents, the plates were allowed to incubate for 10-minute before reading absorbance at 540 nm (Bioteck ELx800 Absorbance Microplate Reader). Plasma NO_2_^−^ concentrations were measured by the same method but omitting the nitrate reductase step. Plasma NO_3_^−^ was calculated as total NO_x_ minus NO_2_^−^. The interassay and intra-assay coefficients of variation were 3.4% and 2.7%, respectively, and the lower limit of detection was 0.24 μM.

#### Plasma CRP

At P21, circulating CRP concentrations were measured by the Biochemistry Assay Laboratory of the Wellcome-MRC Institute of Metabolic Science at Addenbrooke’s Hospital, Cambridge. An in-house dissociation-enhanced lanthanide fluorescence immunoassay (DELFIA) was used using reagents from an R&D Systems DuoSet assay kit (DY1744).^53^ Rat serum samples were diluted 1 in 20 000 in PBS containing 1% BSA (bovine serum albumin) before analysis. The DELFIA assay is a microtitre plate-based time-resolved fluorescence immunoassay. All standards, quality control samples, and unknowns were analyzed in duplicate. Plates were coated with a dilute solution of anti-CRP capture antibody. Results were calculated using the Perkin Elmer Multicalc software package. The assay lower limit of detection was 5 pg/mL.

### Statistical Analysis

Appropriate power calculations derived from previous data sets were performed to determine the minimum sample size required to achieve statistical significance.^12,15,39,42,43^ Wherever possible, procedures were randomized and blinded to experimenters to avoid bias. Postnatal fractional growth rate (FGR) was calculated as the change in body weight over the period of interest, divided by the number of days within that period. For the wire myography analysis, concentration-response curves were analyzed using an agonist-response best-fit line, where the maximal vasomotor response was expressed as percentage of the contraction induced by 76.88 mM K+ (%K_max_ for constriction, maximal relaxation expressed as a percentage of contraction [%R_max_] for relaxation) and the vascular sensitivity was expressed as pD2 (negative logarithm of an EC_50_ [−logEC_50_]). All values are expressed as mean±SEM unless stated. All data were assessed as appropriate using either 2- or 3-way ANOVA (group or treatment) or 2- or 3-way ANOVA with repeated measures comparing the effects of group, dose, or time, in conjunction with the Student-Newman-Keuls post hoc test (Sigma-Stat 3.5; Chicago, IL). For all comparisons, statistical significance was accepted when *P*<0.05.

## Results

### Postnatal Growth, Cardiac and Vascular Histology, and Cardiac Markers of Oxidative Stress

Pups were all born spontaneously on day 22 of gestation. There was no significant difference in birth weight between the groups (control group, 6.54±0.18 g; dexamethasone-treated group, 6.12±0.16 g; dexamethasone and pravastatin–treated group, 6.42±0.13 g; pravastatin-treated group, 6.42±0.15 g). Dexamethasone treatment decreased body weight gain, an effect that was already evident 48 hours following the onset of treatment and continued until P21 (Figure 1). FGR, defined as the increment relative to the first-day measurement of that week, was calculated for individual animals during the first week of life (P0–P7), during the second week of life (P7–P14), and the last week before weaning (P14–P21). In control pups, FGR was highest during P0 to P7 and subsequently decreased during P7 to 14 and P14 to 21 (*P*<0.05, Figure 1C). In contrast, in dexamethasone-treated pups, with or without pravastatin, FGR was the highest during P7 to 14 (*P*<0.05, Figure 1C). However, compared to animals treated with dexamethasone alone, additional administration of pravastatin led to a further increase in FGR between P7 to P14 (*P*<0.05, Figure 1). In pups with only pravastatin, the FGR pattern was similar than controls.

Compared to control animals, dexamethasone-treated pups with or without pravastatin had significantly shorter crown-rump length, and a significant increase in the ponderal index, the brain:liver weight ratio, and the head diameter:body weight ratio, all indices reflecting asymmetric growth (Table 1). Pravastatin treatment alone did not affect any of these variables (Table 1). Compared with organs from control animals, the absolute heart and liver weights were lower, and the relative brain and lung weights were greater, in dexamethasone-treated animals (Table 1). The reduction in heart weight appeared to be due to a fall in the weight of the LV, but not the right ventricle, in dexamethasone-treated pups; an effect consistent with a fall in the LV, but not the right ventricle, wall area (Table 1 and Figure S1). Concomitant treatment of dexamethasone groups with pravastatin improved the absolute heart weight but it did not affect any other organ measure. Pravastatin treatment alone did not affect any of these variables (Table 1). No treatment had any effect on the morphology of the thoracic aorta, or on markers of cardiac oxidative stress (Table 1).

**Table 1.**
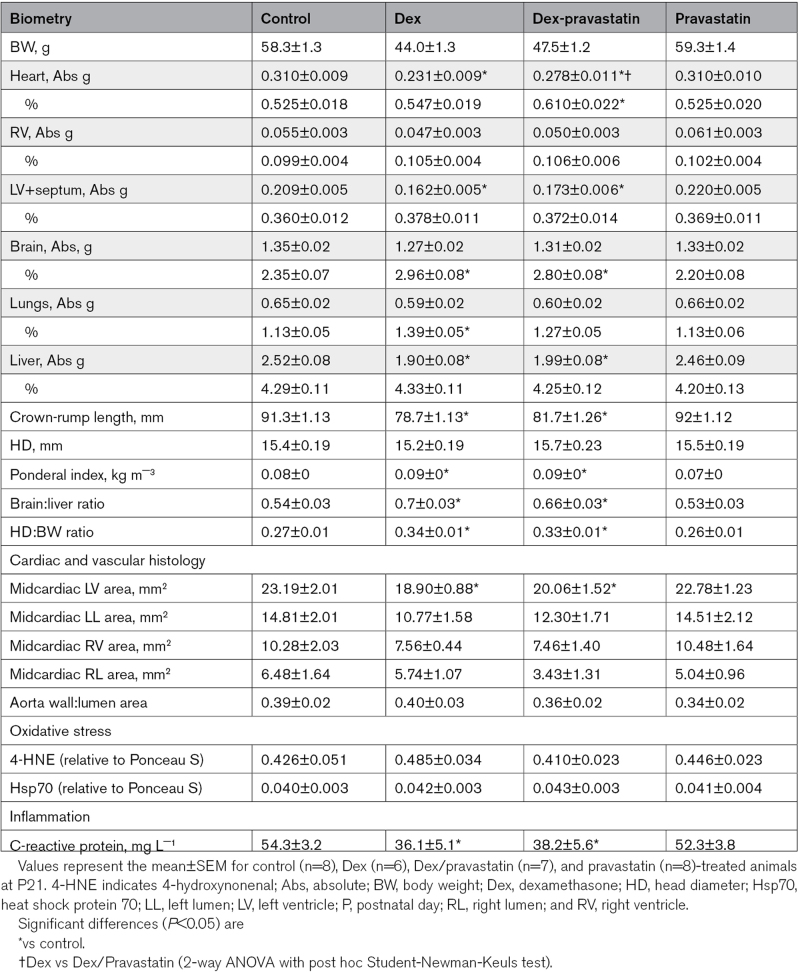
Biometry, Cardiovascular Histology, and Circulating Agents

### In Vivo Cardiovascular Function and Circulating Agents

**Figure 2.**
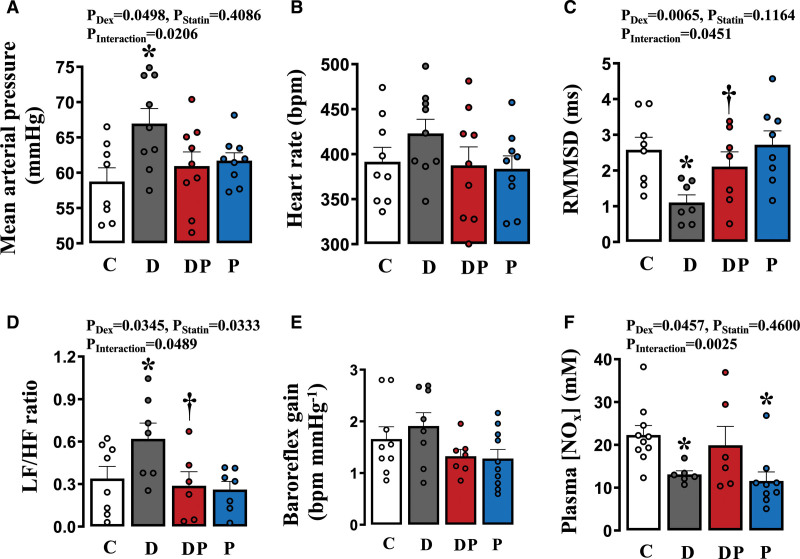
**In vivo cardiovascular function and NO bioavailability.** Values are mean± SEM for basal mean arterial pressure (**A**), heart rate (**B**), heart rate variability in the time (RMMSD), and frequency (low frequency [LF]/ high frequency [HF] ratio) domains (**C** and **D**, respectively), baroreflex gain (**E**) and circulating plasma NO_x_ (NO species; nitrates and nitrites; **F**) in P21 pups from control (, C, n=8), dexamethasone (Dex)-treated group (, D, n=9), Dex and pravastatin–treated group (, DP, n=9) and pravastatin-treated group (, P, n=9) treatment groups. The *P* values for the main factors and interaction in the 2-way ANOVA are shown. Significant differences (*P*<0.05) for the post hoc Student-Newman-Keuls are *vs control. †D vs DP.

At P21, in control animals, values for basal arterial blood pressure and heart rate were 60±2 mm Hg and 392±16 bpm, respectively, and were similar to previously published measurements in age-matched Wistar rat pups under urethane anesthesia^36^ (Figure 2A and 2B). Postnatal treatment with dexamethasone led to a significant increase in arterial pressure (Figure 2A). Concomitant treatment with pravastatin in pups receiving dexamethasone restored arterial basal blood pressure to control levels (Figure 2A). Pravastatin treatment alone had no effect on basal cardiovascular variables. When determining changes in heart rate variability at P21, in the time domain, dexamethasone-treated pups displayed a lower square root of the mean of the sum of the squares of difference between adjacent NN intervals, an indicator of parasympathetic tone (Figure 2C). In the frequency domain, dexamethasone led to an increase in the low-to-high frequency ratio, an indicator of sympathetic dominance (Figure 2D). Concomitant treatment with pravastatin in dexamethasone-treated pups prevented the changes in both the time and frequency domains of heart rate variability (Figure 2C and D). Pravastatin treatment alone had no effect on arterial blood pressure, heart rate, or heart rate variability (Figure 2A through 2D).

At P21, circulating plasma levels of NO_x_ (nitrates and nitrites) were 22.3±2.2 μM in control pups and were similar to values previously reported in rodents.^35^ Postnatal treatment with dexamethasone led to a significant fall in plasma NO_x_ at P21, whereas this was prevented with concomitant postnatal pravastatin treatment (Figure 2E). Postnatal pravastatin treatment alone also significantly decreased plasma NO_x_ levels compared to control pups at P21 (Figure 2E). At P21, relative to control animals, circulating levels of CRP, a marker of inflammation, were similarly reduced in dexamethasone-treated pups with or without pravastatin (Table 1). Pravastatin treatment alone had no effect on plasma concentrations of CRP (Table 1).

### Isolated Cardiac Function

**Figure 3.**
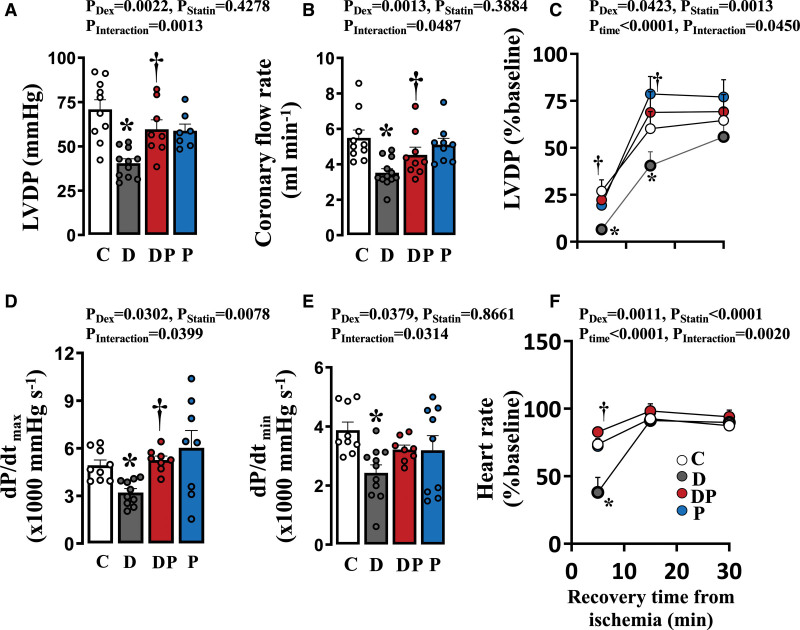
**Isolated cardiac function.** Values are the mean±SEM for left ventricular developed pressure (LVDP; **A**), coronary flow rate (**B**), the LVDP following 15-minute ischemia (**C**), the maximum first derivatives of the left ventricular pressure with respect to time (dP/dt_max_; **D**); the minimum first derivatives of the left ventricular pressure with respect to time (dP/dt_min_; **E**); and the heart rate following 15-minute ischemia (**F**) in Langendorff preparations from control (, C, n=7), dexamethasone (Dex)-treated group (, D, n=7), Dex and pravastatin–treated group (, DP, n=7) and pravastatin-treated group (, P, n=7) treated animals. The *P* values for the main factors and interaction in the 2- or 3-way ANOVA are shown. Significant differences (*P*<0.05) for the post hoc Student-Newman-Keuls are *vs control. †D vs DP.

At P21, isolated hearts from dexamethasone-treated pups displayed lower basal LV developed pressure, coronary flow rates, dP/dt_max_, and dP/dt_min_ when compared to control pups (Figure 3A through 3D). Concomitant treatment with pravastatin restored all these variables toward control values (Figure 3A through 3D). Following ischemia, within the first 10 minutes of recovery, hearts from dexamethasone-treated animals displayed lower values for LV-developed pressure and heart rate compared with control animals. However, values were comparable to control animals by 30 minutes postischemia (Figure 3E and 3F). Again, concomitant pravastatin protected against the dexamethasone-induced lower LV developed pressure and heart rate following ischemia (Figure 3E and 3F). Pravastatin treatment alone did not affect basal or postischemia cardiac function (Figure 3E and 3F).

### Isolated Peripheral Vascular Reactivity

**Figure 4.**
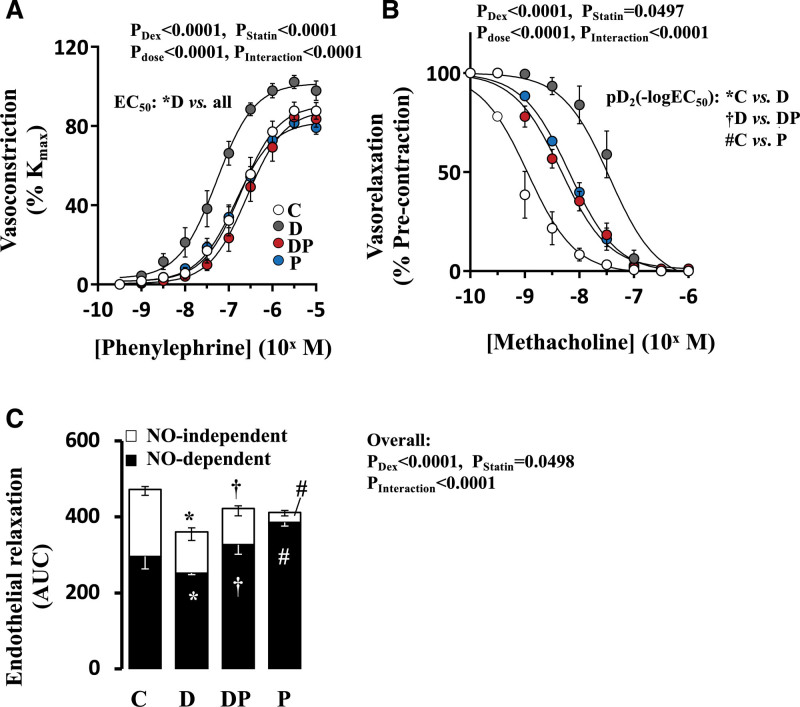
**Isolated peripheral vascular reactivity.** Values are the mean±SEM for the dose-response constrictor curve to phenylephrine (**A**), the dose-response dilator curve to methacholine following preconstriction (**B**), and the nitric oxide (NO)-dependent and NO-independent vasodilator components to the methacholine-induced relaxation in femoral arteries isolated from control group (, C, n=8), dexamethasone (Dex)-treated group (, D, n=7), Dex and pravastatin–treated group (, DP, n=7) and pravastatin-treated group (, P, n=7) treated animals. The *P* values for the main factors and interaction in the 2- or 3-way ANOVA are shown. For **A** and **B**, significant differences (*P*<0.05) for the post hoc Student-Newman-Keuls are also detailed on the figures. For **C**, significant differences (*P*<0.05) for the post hoc Student-Newman-Keuls are *vs control. †D vs DP, #C vs P. AUC indicates area under the curve; and pD2(−logEC_50_), negative logarithm of an EC_50_.

At P21, dexamethasone treatment promoted a leftward shift in the concentration-response constrictor curve to increasing doses of phenylephrine, showing an increase in α_1_-adrenergic sensitivity in isolated femoral arteries (Figure 4A). Concomitant pravastatin treatment restored the shift back to control values (Figure 4A). Pravastatin treatment alone had no significant effect on phenylephrine-induced contraction in femoral arteries at P21 (Figure 4A). Endothelial-dependent dilatation, assessed by application of methacholine, revealed markedly impaired relaxation in dexamethasone-treated animals, showing a pronounced rightward shift in the concentration-response curve relative to controls (Figure 4B). Concomitant pravastatin treatment partially restored the vasodilator response toward control values (Figure 4B). In this case, pravastatin treatment alone also impaired the relaxant response to methacholine, still showing a significant rightward shift in the concentration-response curve relative to controls (Figure 4B). Detailed analysis of NO-dependent and NO-independent components of the endothelial-dependent relaxation revealed that NO-dependent dilator reactivity was significantly decreased in dexamethasone-treated pups (Figure 4C). Concomitant pravastatin treatment restored the NO-dependent relaxation towards control values (Figure 4C). Relative to control, pravastatin treatment alone also significantly enhanced the NO-dependent and significantly decreased the NO-independent components of the methacholine-induced relaxation in isolated femoral arteries (Figure 4C).

### Pulmonary Maturation

**Figure 5.**
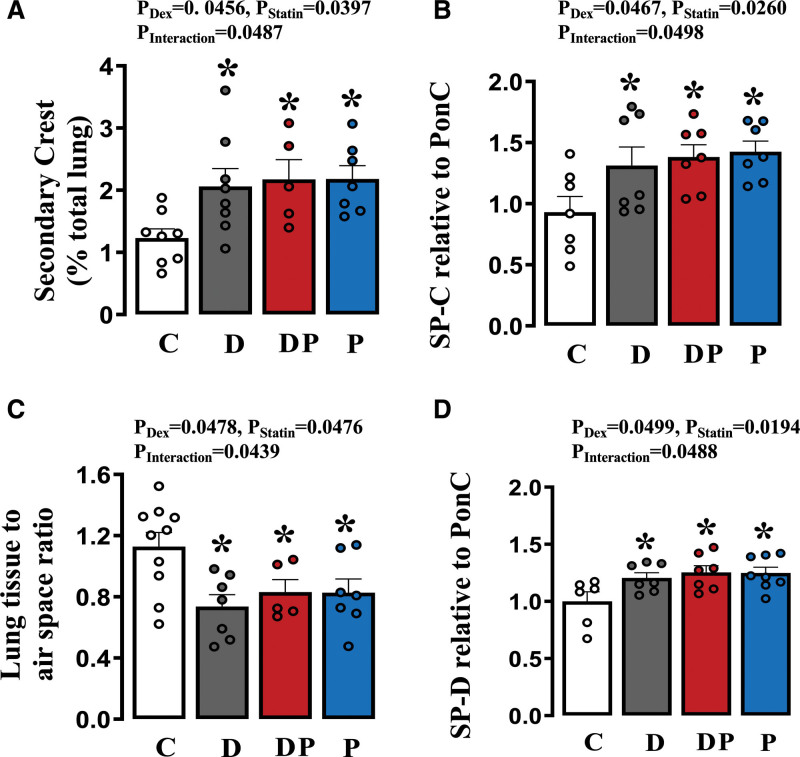
**Lung structure and surfactant expression.** Values are the mean±SEM for secondary crest formation (**A**), lung tissue to air space ratio (**B**), pulmonary tissue expression of SP-C (surfactant protein C; **C**), and pulmonary tissue expression of SP-D (surfactant protein D; **D**) in control group (, C, n=7), dexamethasone (Dex)-treated group (, D, n=7), Dex and pravastatin–treated group (, DP, n=7) and pravastatin-treated group (, P, n=7) animals. The *P* values for the main factors and interaction in the 2-way ANOVA are shown. Significant differences (*P*<0.05) for the post hoc Student-Newman-Keuls are *vs control. PonC indicates ponceau.

Relative to lungs of control animals, those from dexamethasone-treated pups showed a greater formation of secondary crests, an increased protein expression of surfactant C and D, and a fall in the lung tissue: air space ratio; all indices of pulmonary maturation (Figure 5A through 5D). Concomitant pravastatin treatment to dexamethasone groups did not affect these changes (Figure 5A through 5D). Relative to lungs of control animals, those from pups treated with pravastatin alone also showed a greater formation of secondary crests, an increased expression of surfactant, and a fall in the lung tissue: air space ratio (Figure 5A through 5D).

## Discussion

The newborn rat is an established preclinical model of human prematurity, as developmental landmarks in many physiological systems in the first week of postnatal life in this species compared with prenatal milestones in the human.^54,55^ The data show that treating newborn rat pups with dexamethasone, adopting a human clinically relevant course used to treat prematurity, stunts growth, and increases the risk of hypertension, cardiac remodeling, and cardiovascular dysfunction later in life. Combined treatment of the newborn pup with dexamethasone and pravastatin normalizes all cardiovascular adverse side-effects while having a mild protective effect on postnatal growth rate, without inhibiting beneficial effects of glucocorticoids on indices of lung maturation and inflammation. Mechanisms underlying the protective effects of pravastatin in dexamethasone-treated animals include an increase in NO bioavailability and signaling, as well as restoration of sympathetic hyper-reactivity in the heart and vasculature. The data also show that treatment of newborn pups with pravastatin alone accelerates indices of lung maturation and impairs endothelial function in peripheral resistance arteries. Therefore, the data support the hypothesis tested that combined dexamethasone and pravastatin therapy is safer than dexamethasone alone in the treatment of preterm birth.

The effects of perinatal exposure to excess glucocorticoids in increasing arterial blood pressure in the fetal and newborn periods^8,11,56–60^ and in programming hypertension in later life are well known.^61–63^ In the present study, postnatal dexamethasone treatment also led to significant elevations in basal arterial blood pressure when measured at weaning. Given that arterial blood pressure is the product of cardiac output and peripheral vascular resistance, we established effects on vasomotor reactivity in the peripheral vasculature. Femoral vessel isolated from dexamethasone-treated pups had markedly enhanced constrictor responses to the α_1_-adrenergic agonist phenylephrine, while significantly impaired dilator responses to the endothelium-dependent agonist methacholine. Deeper analysis revealed a depressive effect of dexamethasone on the NO-dependent component of endothelium-mediated relaxation in the peripheral vessels. Further, treatment of pups with dexamethasone did not induce markers of oxidative stress but it led to a fall in circulating NO bioavailability. Consistent with available literature,^59–61^ these data support a shift towards a peripheral vasoconstrictor phenotype, resulting from impaired NO signaling and sympathetic dominance, promoting the increase in arterial blood pressure in dexamethasone-treated pups. Additional data reported in the present study on heart rate variability further underpin an effect of dexamethasone promoting sympathetic dominance. These results compliment other recent work where antenatal betamethasone also led to a decrease in the square root of the mean of the sum of the squares of difference between adjacent NN intervals and a fall in heart rate variability in 1.8-year-old lambs.^64^ In the present study, the results also show that concomitant treatment with pravastatin in dexamethasone-treated pups restored towards control values circulating plasma NO_x_, the peripheral vasculature NO-dependent dilator reactivity, the peripheral vasculature α_1_-adrenergic constrictor reactivity, as well as the alterations in heart rate variability. Integrating these findings, the data, therefore, support the concept that glucocorticoid therapy in the newborn period impairs the developing cardiovascular function by decreasing NO bioavailability and signaling, which normally acts to oppose sympathetic outflow and hyper-reactivity, and that pravastatin, by restoring NO bioavailability, normalizes the adverse effects of dexamethasone on the cardiovascular system. In support, atorvastatin during pregnancy restored endothelial function in adult rats exposed to a protein-restricted diet during pregnancy.^65^ Furthermore, pravastatin ameliorated fetal cardiac dysfunction in a pregnancy model of glucocorticoid excess.^66^ This suggests that depletion in NO bioavailability may represent a common mechanism underlying the programming of vascular dysfunction by excess glucocorticoids or maternal malnutrition and that statins provide a potential intervention.

Although hypertensive effects of dexamethasone may be welcome in the maintenance of arterial blood pressure of the preterm infant in the neonatal intensive care unit, excessive and sustained increases in blood pressure above basal values can be dangerous, as they may affect cardiac function and trigger remodeling. Additional data presented in this study show that hearts of weanling pups treated with dexamethasone during the newborn period were weaker, showing impaired contractility, left ventricular wall thinning, reduced coronary flow rate, and delayed recovery to an ischemic challenge. These data are consistent with the findings of Bal et al^14^ who reported an increase in systolic dysfunction in adult rats treated with dexamethasone during the neonatal period. Although an increase in arterial blood pressure promotes cardiac hypertrophy in the first instance, sustained increase in cardiac afterload may overwhelm myocardial compensation and switch remodeling towards ventricular thinning, reminiscent of dilated cardiomyopathy.^16,67^ Consequently, combined treatment of pravastatin with dexamethasone, by maintaining NO bioavailability and signaling and restoring peripheral vascular resistance, may alleviate cardiac afterload and its adverse consequences on cardiac structure and function.

In the human literature, the negative effects on postnatal growth of synthetic glucocorticoid exposure during the perinatal period are well documented. For instance, following neonatal glucocorticoid therapy, height and more so weight are significantly decreased in school-age children.^9^ Such findings are also widely supported in preclinical animal models,^10,15,59,60^ and the growth-stunting effects of dexamethasone are mediated by antiproliferative effects during development, switching tissue accretion to differentiation.^68^ In the present study, the growth restriction induced by dexamethasone was also asymmetric, reflected by an increase in the ponderal index, in the brain: liver weight ratio, and in the head diameter: body weight ratio.

In pups treated concomitantly with pravastatin, the dexamethasone-induced repression of growth rate was mildly improved between 7 and 14 days of postnatal life, but body weight was not restored to control levels by weaning. This implies that while increasing NO bioavailability may improve growth, for instance via improving perfusion and the delivery of oxygen and nutrients and/or promoting angiogenesis,^69,70^ these effects are not significant enough to revert the powerful stunting effects of steroids in the postnatal period. It is possible that some of the effects of dexamethasone promoting cardiac wall thinning are also direct, due to similar inhibitory effects of glucocorticoids on cardiomyocyte proliferation during development.^71^ In this regard, it is of interest that statins have been reported to protect cardiomyocyte formation.^72–74^ For example, atorvastatin protects cardiac progenitor cells from hypoxia-induced cell growth inhibition.^75^ In the present study, since pravastatin protected against dexamethasone-induced cardiovascular dysfunction but not against dexamethasone-induced growth restriction, growth restriction per se appears not be indispensable for increasing the risk of cardiovascular dysfunction later in life.

In clinical practice today, there is considerable debate surrounding the choice and dosing strategies of glucocorticoid therapy in preterm infants.^8,11^ Hydrocortisone may be used preferentially over dexamethasone as it has fewer adverse side-effects.^1^ Conversely, dexamethasone compared with hydrocortisone is known to confer greater benefit on respiratory function, including earlier extubation, and decreased dependence on O_2_ supplementation.^8,11^ Greater detrimental and beneficial effects of dexamethasone over hydrocortisone are understandable given its much greater relative potency and longer half-life.^76^ In clinical practice, the greater beneficial effects on respiratory function of dexamethasone over hydrocortisone would therefore make it the glucocorticoid of choice, as long as its detrimental effects could be tamed without affecting its beneficial effects on the lung. Data in the present study show that combined treatment of pups in the neonatal period with dexamethasone and statins diminishes the unwanted side-effects of the glucocorticoid on the cardiovascular system while maintaining lung maturational and anti-inflammatory effects.

Interestingly, in the present study, neonatal treatment with pravastatin alone also had some significant effects. It decreased circulating NOx concentrations, reduced endothelium-dependent relaxation, and promoted indices of lung maturation. Although treatment of dexamethasone-exposed pups with pravastatin may replenish circulating NO back to baseline, treatment with pravastatin in control pups when basal levels of circulating NO are normal may induce negative feedback. In the present study, this may explain the reduction in plasma NOx and in endothelium-dependent relaxation in peripheral resistance arteries in pups treated with pravastatin alone. These data are consistent with a similar depressive effect of vitamin C treatment alone in rat pups on circulating NOx and endothelial function in peripheral resistance arteries.^77^ The antioxidant actions of vitamin C also increase NO bioavailability and signaling.^78^ It has also been reported that statins can inhibit NO-independent pathways, such as prostanoid and endothelium-derived hyperpolarizing factor (EDHF).^79,80^ The mechanisms underlying the promotion of indices of lung maturation by pravastatin treatment alone in the present study are not known. However, we have recently reported similar lung maturational effects in the offspring of vitamin C treatment in ovine pregnancy.^81^ Therefore, the mechanism via which an increase in NO bioavailability, resulting from antioxidant or statin treatment, promotes lung maturation is clearly a rich avenue of future research.

The study has important limitations. Although the term newborn rat pup resembles the human preterm fetus when considering temporal milestones of cardiovascular development, a clear next step is to determine whether these data can be translated to established preclinical models in animals born preterm, like fetal sheep delivered at 0.7 of gestation. A second limitation is that in vivo cardiovascular function was determined in the anesthetized rat pup at weaning. Future experiments in sheep born preterm should resolve this by investigating cardiovascular function in the conscious chronically instrumented animal, following full postsurgical recovery. Such experiments should also be extended to determine cardiovascular function in the adult animal, to address which effects measured at weaning, persist, reverse, or amplify with time. A further limitation of the study is that outcomes were measured only in male pups to control for, but not to address, sex differences. Challenges during development are known to have sexually dimorphic effects.^82^ Therefore, future studies should also be completed in male as well as female offspring.

In summary, this study in rats shows that a human clinically relevant course of dexamethasone used in the treatment of the preterm infant promotes adverse effects on cardiovascular structure and function when measured at weaning. Combined treatment of dexamethasone with pravastatin protects against deleterious effects on the cardiovascular system while maintaining beneficial effects of dexamethasone on lung maturation and inflammation. Therefore, combined dexamethasone and pravastatin may be safer in the treatment of preterm birth.

### Perspectives

In clinical practice, synthetic glucocorticoids are not only used as therapy for preterm infants but also to treat pregnant women threatened with preterm birth. In this case, maternal intramuscular injection with dexamethasone or betamethasone is designed to cross the placenta, reach the fetal circulation, and accelerate fetal lung maturation. Antenatal glucocorticoid treatment is well-established worldwide and it is one of the best examples of the successful translation of basic science research into lifesaving clinical intervention. Despite this, there are also growing concerns about its safety as accumulating evidence suggests adverse effects of the therapy on the cardiovascular system of the offspring. Therefore, there is also an urgent need to fine-tune antenatal glucocorticoid therapy and make it safer for the treatment of preterm birth. Extending the findings of the present study to antenatal therapy, combined antenatal glucocorticoid and statin treatment in pregnancy threatened with preterm birth may maintain beneficial effects of glucocorticoids on the fetal lung while protecting the developing cardiovascular system and preventing a fetal origin of future cardiovascular risk. Therefore, combined antenatal glucocorticoid and statin therapy may again be safer for the treatment of pregnancy threatened with preterm birth. However, this perspective needs to be tested.

## Article Information

### Acknowledgments

We are grateful to the staff of the University of Cambridge Biological Services for helping with the maintenance of the animals. D.A. Giussani is a Fellow of the Lister Institute for Preventive Medicine and a Royal Society Wolfson Research Merit Award Holder. We would like to acknowledge the support from Professor Fred K. Lotgering and Deirdre P. Murphy for help with some of the lung histology.

### Author Contributions

D.A. Giussani, J.B. Derks, and F.K. Lotgering contributed to Conceptualization. A.D. Kane, E.A. Herrera, Y. Niu, E.J. Camm, B.J. Allison, D. Tijsseling, C. Lusby, K.L. Brain, I.M. Bronckers, C.M. Cross, D.P. Murphy, and L. Berends contributed to Methodology. A.D. Kane, E.A. Herrera, Y. Niu, E.J. Camm, B.J. Allison, D. Tijsseling, C. Lusby, K.L. Brain, I.M. Bronckers, C.M. Cross, D.P. Murphy, L. Berends, and D.A. Giussani contributed to Data and Statistical Analysis. A.D. Kane, E.A. Herrera, and D.A. Giussani contributed to Writing – Original Draft. A.D. Kane, E.A. Herrera, and D.A. Giussani contributed to Writing – Review & Editing. A.D. Kane, Y. Niu, E.A. Herrera, E.J. Camm, B.J. Allison, D. Tijsseling, C. Lusby, K.L. Brain, I.M. Bronckers, C.M. Cross, D.P. Murphy, L. Berends, and D.A. Giussani contributed to Visualization. D.A. Giussani, J.B. Derks, and F.K. Lotgering contributed to Supervision. D.A. Giussani, J.B. Derks, and F.K. Lotgering contributed to Project Administration. D.A. Giussani and J.B. Derks contributed to Funding Acquisition.

### Sources of Funding

A.D. Kane was supported by the Frank Edward Elmore Fund and the James Baird Fund. This study was funded by the British Heart Foundation and the BBSRC.

### Disclosures

None.

## Supplementary Material



## References

[1] Halliday H Ehrenkranz R Doyle L. Early (< 8 days) postnatal corticosteroids for preventing chronic lung disease in preterm infants. Cochrane Database Syst Rev. 2010;1:CD001146. doi: 10.1002/14651858.CD001146.pub2 10.1002/14651858.CD001146.pub3 20091516

[2] Northway WH Jr Rosan RC Porter DY. Pulmonary disease following respirator therapy of hyaline-membrane disease: bronchopulmonary dysplasia. N Engl J Med. 1967;276:357–368. doi: 10.1056/nejm196702162760701 5334613 10.1056/NEJM196702162760701

[3] Liggins GC Howie RN. A controlled trial of antepartum glucocorticoid treatment for prevention of the respiratory distress syndrome in premature infants. Pediatrics. 1972;50:515–525. doi: 10.1542/peds.50.4.515 4561295

[4] Liggins G. Premature delivery of foetal lambs infused with glucocorticoids. J Endocrinol. 1969;45:515–523. doi: 10.1677/joe.0.0450515 5366112 10.1677/joe.0.0450515

[5] Cummings JJ D’Eugenio DB Gross SJ. A controlled trial of dexamethasone in preterm infants at high risk for bronchopulmonary dysplasia. N Engl J Med. 1989;320:1505–1510. doi: 10.1056/nejm198906083202301 2657423 10.1056/NEJM198906083202301

[6] Ballard PL Ballard RA. Scientific basis and therapeutic regimens for use of antenatal glucocorticoids. Am J Obstet Gynecol. 1995;173:254–262. doi: 10.1016/0002-9378(95)90210-4 7631700 10.1016/0002-9378(95)90210-4

[7] Roberts D Dalziel S. Antenatal corticosteroids for accelerating fetal lung maturation for women at risk of preterm birth. Cochrane Database Syst Rev. 2006;3:CD004454. doi: 10.1002/14651858.CD004454.pub2 10.1002/14651858.CD004454.pub2 16856047

[8] Doyle LW Ehrenkranz RA Halliday HL. Dexamethasone treatment in the first week of life for preventing bronchopulmonary dysplasia in preterm infants: a systematic review. Neonatology. 2010;98:217–224. doi: 10.1159/000286210 20389126 10.1159/000286210

[9] Yeh TF Lin YJ Lin HC Huang CC Hsieh WS Lin CH Tsai CH. Outcomes at school age after postnatal dexamethasone therapy for lung disease of prematurity. N Engl J Med. 2004;350:1304–1313. doi: 10.1056/nejmoa032089 15044641 10.1056/NEJMoa032089

[10] de Vries A Holmes MC Heijnis A Seier JV Heerden J Louw J Wolfe-Coote S Meaney MJ Levitt NS Seckl JR. Prenatal dexamethasone exposure induces changes in nonhuman primate offspring cardiometabolic and hypothalamic-pituitary-adrenal axis function. J Clin Invest. 2007;117:1058–1067. doi: 10.1172/JCI30982 17380204 10.1172/JCI30982 PMC1821070

[11] Doyle LW Ehrenkranz RA Halliday HL. Dexamethasone treatment after the first week of life for bronchopulmonary dysplasia in preterm infants: a systematic review. Neonatology. 2010;98:289–296. doi: 10.1159/000286212 20453523 10.1159/000286212

[12] Herrera EA Verkerk MM Derks JB Giussani DA. Antioxidant treatment alters peripheral vascular dysfunction induced by postnatal glucocorticoid therapy in rats. PLoS One. 2010;5:e9250. doi: 10.1371/journal.pone.0009250 20174656 10.1371/journal.pone.0009250 PMC2822858

[13] de Vries W Van Der F Bakker J Kamphuis P Van Oosterjout M Schipper M Smid G Bartelds B Van Bel F. Alterations in adult rat heart after neonatal dexamethasone therapy. Pediatr Res. 2002;52:900–906. doi: 10.1203/00006450-200212000-00015 12438668 10.1203/00006450-200212000-00015

[14] Bal M de Vries W van der Leij F van Oosterhout M Berger R Baan J van der Wall E van Bel F Steendijk P. Neonatal glucocorticosteroid treatment causes systolic dysfunction and compensatory dilation in early life: studies in 4-week-old prepubertal rats. Pediatr Res. 2005;58:46–52. doi: 10.1203/01.PDR.0000163617.01673.9A 15985685 10.1203/01.PDR.0000163617.01673.9A

[15] Adler A Camm E Hansell J Richter H Giussani DA. Investigation of the use of antioxidants to diminish the adverse effects of postnatal glucocorticoid treatment on mortality and cardiac development. Neonatology. 2010;98:73–83. doi: 10.1159/000275561 20068362 10.1159/000275561

[16] Bal MP de Vries WB Steendijk P Homoet-van der Kraak P van der Leij FR Baan J van Oosterhout MF van Bel F. Histopathological changes of the heart after neonatal dexamethasone treatment: studies in 4-, 8-, and 50-week-old rats. Pediatr Res. 2009;66:74–79. doi: 10.1203/PDR.0b013e3181a283a0 19287345 10.1203/PDR.0b013e3181a283a0

[17] Iuchi T Akaike M Mitsui T Ohshima Y Shintani Y Azuma H Matsumoto T. Glucocorticoid excess induces superoxide production in vascular endothelial cells and elicits vascular endothelial dysfunction. Circ Res. 2003;92:81–87. doi: 10.1161/01.res.0000050588.35034.3c 12522124 10.1161/01.res.0000050588.35034.3c

[18] Zhang Y Croft KD Mori TA Schyvens CG McKenzie KU Whitworth JA. The antioxidant tempol prevents and partially reverses dexamethasone-induced hypertension in the rat. Am J Hypertens. 2004;17:260–265. doi: 10.1016/j.amjhyper.2003.11.004 15001201 10.1016/j.amjhyper.2003.11.004

[19] Wallerath T Witte K Schäfer SC Schwarz PM Prellwitz W Wohlfart P Kleinert H Lehr H-A Lemmer B Förstermann U. Down-regulation of the expression of endothelial NO synthase is likely to contribute to glucocorticoid-mediated hypertension. Proc Natl Acad Sci USA. 1999;96:13357–13362. doi: 10.1073/pnas.96.23.13357 10557325 10.1073/pnas.96.23.13357 PMC23952

[20] Simmons WW Ungureanu-Longrois D Smith GK Smith TW Kelly RA. Glucocorticoids regulate inducible nitric oxide synthase by inhibiting tetrahydrobiopterin synthesis and L-arginine transport. J Biol Chem. 1996;271:23928–23937. doi: 10.1074/jbc.271.39.23928 8798625 10.1074/jbc.271.39.23928

[21] Morrison S Gardner DS Fletcher AJW Bloomfield MR Giussani DA. Enhanced nitric oxide activity offsets peripheral vasoconstriction during acute hypoxaemia via chemoreflex and adrenomedullary actions in the sheep fetus. J Physiol. 2003;547:283–291. doi: 10.1113/jphysiol.2002.032615 12562956 10.1113/jphysiol.2002.032615 PMC2342630

[22] Gardner DS Fowden AL Giussani DA. Adverse intrauterine conditions diminish the fetal defense against acute hypoxia by increasing nitric oxide activity. Circulation. 2002;106:2278–2283. doi: 10.1161/01.cir.0000033827.48974.c8 12390960 10.1161/01.cir.0000033827.48974.c8

[23] Schultz HD. Nitric oxide regulation of autonomic function in heart failure. Curr Heart Fail Rep. 2009;6:71–80. doi: 10.1007/s11897-009-0012-x 19486590 10.1007/s11897-009-0012-x PMC2804940

[24] Steinberg D. The statins in preventive cardiology. N Engl J Med. 2008;359:1426–1427. doi: 10.1056/nejmp0806479 18832243 10.1056/NEJMp0806479

[25] Maki-Petaja K Wilkinson I. Anti-inflammatory drugs and statins for arterial stiffness reduction. Curr Pharm Des. 2009;15:290–303. doi: 10.2174/138161209787354221 19149619 10.2174/138161209787354221

[26] Glynn RJ Danielson E Fonseca FAH Genest J Gotto AM Jr Kastelein JJP Koenig W Libby P Lorenzatti AJ MacFadyen JG. A randomized trial of rosuvastatin in the prevention of venous thromboembolism. N Engl J Med. 2009;360:1851–1861. doi: 10.1056/NEJMoa0900241 19329822 10.1056/NEJMoa0900241 PMC2710995

[27] Horwich TB Middlekauff HR. Potential autonomic nervous system effects of statins in heart failure. Heart Fail Clin. 2008;4:163–170. doi: 10.1016/j.hfc.2008.01.004 18433696 10.1016/j.hfc.2008.01.004 PMC2440345

[28] Gelosa P Cimino M Pignieri A Tremoli E Guerrini U Sironi L. The role of HMG-CoA reductase inhibition in endothelial dysfunction and inflammation. Vasc Health Risk Manag. 2007;3:567–577. 18078008 PMC2291301

[29] Blum A Shamburek R. The pleiotropic effects of statins on endothelial function, vascular inflammation, immunomodulation and thrombogenesis. Atherosclerosis. 2009;203:325–330. doi: 10.1016/j.atherosclerosis.2008.08.022 18834985 10.1016/j.atherosclerosis.2008.08.022

[30] Adam O Laufs U. Antioxidant effects of statins. Arch Toxicol. 2008;82:885–892. doi: 10.1007/s00204-008-0344-4 18670762 10.1007/s00204-008-0344-4

[31] Laufs U La Fata V Plutzky J Liao JK. Upregulation of endothelial nitric oxide synthase by HMG CoA reductase inhibitors. Circulation. 1998;97:1129–1135. doi: 10.1161/01.cir.97.12.1129 9537338 10.1161/01.cir.97.12.1129

[32] Kaesemeyer WH Caldwell RB Huang J Caldwell RW. Pravastatin sodium activates endothelial nitric oxide synthase independent of its cholesterol-lowering actions. J Am Coll Cardiol. 1999;33:234–241. doi: 10.1016/s0735-1097(98)00514-2 9935036 10.1016/s0735-1097(98)00514-2

[33] Gilbert JS Nijland MJ. Sex differences in the developmental origins of hypertension and cardiorenal disease. Am J Physiol Regul Integr Comp Physiol. 2008;295:R1941–R1952. doi: 10.1152/ajpregu.90724.2008 18971349 10.1152/ajpregu.90724.2008 PMC2685301

[34] Roghair RD Segar JL Volk KA Chapleau MW Dallas LM Sorenson AR Scholz TD Lamb FS. Vascular nitric oxide and superoxide anion contribute to sex-specific programmed cardiovascular physiology in mice. Am J Physiol Regul Integr Comp Physiol. 2009;296:R651–R662. doi: 10.1152/ajpregu.90756.2008 19144750 10.1152/ajpregu.90756.2008 PMC2665850

[35] Mondo CK Yang W-S Zhang N Huang T-G. Anti-oxidant effects of atorvastatin in dexamethasone-induce hypertension in the rat. Clin Exp Pharmacol Physiol. 2006;33:1029–1034. doi: 10.1111/j.1440-1681.2006.04482.x 17042910 10.1111/j.1440-1681.2006.04482.x

[36] Kasparov S Paton JFR. Changes in baroreceptor vagal reflex performance in the developing rat. Pflugers Arch. 1997;434:438–444. doi: 10.1007/s004240050418 9211810 10.1007/s004240050418

[37] Arnold AC Isa K Shaltout HA Nautiyal M Ferrario CM Chappell MC Diz DI. Angiotensin-(1–12) requires angiotensin converting enzyme and AT1 receptors for cardiovascular actions within the solitary tract nucleus. Am J Physiol Heart Circ Physiol. 2010;299:H763–H771. doi: 10.1152/ajpheart.00345.2010 20562338 10.1152/ajpheart.00345.2010 PMC2944473

[38] Peotta VA Vasquez EC Meyrelles SS. Cardiovascular neural reflexes in L-NAME–induced hypertension in mice. Hypertension. 2001;38:555–559. doi: 10.1161/01.hyp.38.3.555 11566930 10.1161/01.hyp.38.3.555

[39] Electrophysiology Task Force of the European Society of Cardiology the North American Society of Pacing. Heart rate variability: standards of measurement, physiological interpretation, and clinical use. Circulation. 1996;93:1043–1065 doi: 10.1161/01.CIR.93.5.1043 8598068

[40] Kane AD Herrera EA Camm EJ Giussani DA. Vitamin C prevents intrauterine programming of in vivo cardiovascular dysfunction in the rat. Circ J. 2013;77:2604–2611. doi: 10.1253/circj.cj-13-0311 23856654 10.1253/circj.cj-13-0311

[41] Rowan W Campen M Wichers L Watkinson W. Heart rate variability in rodents: uses and caveats in toxicological studies. In: Cardiovascular Toxicology. Humana Press Inc.; 2007:28–51. 10.1007/s12012-007-0004-6 17646680

[42] Niu Y Kane AD Lusby CM Allison BJ Chua YY Kaandorp JJ Nevin-Dolan R Ashmore TJ Blackmore HL Derks JB. Maternal allopurinol prevents cardiac dysfunction in adult male offspring programmed by chronic hypoxia during pregnancy. Hypertension. 2018;72:971–978. doi: 10.1161/hypertensionaha.118.11363 30354714 10.1161/HYPERTENSIONAHA.118.11363 PMC6135482

[43] Niu Y Herrera EA Evans RD Giussani DA. Antioxidant treatment improves neonatal survival and prevents impaired cardiac function at adulthood following neonatal glucocorticoid therapy. J Physiol. 2013;591:5083–5093. doi: 10.1113/jphysiol.2013.258210 23940378 10.1113/jphysiol.2013.258210 PMC3810811

[44] Gage GJ Kipke DR Shain W. Whole animal perfusion fixation for rodents. J Vis Exp. 2012;65:3564. doi: 10.3791/3564 10.3791/3564 PMC3476408 22871843

[45] Itani N Skeffington KL Beck C Niu Y Giussani DA. Melatonin rescues cardiovascular dysfunction during hypoxic development in the chick embryo. J Pineal Res. 2016;60:16–26. doi: 10.1111/jpi.12283 26444711 10.1111/jpi.12283 PMC4832387

[46] Itani N Skeffington KL Beck C Niu Y Katzilieris-Petras G Smith N Giussani DA. Protective effects of pravastatin on the embryonic cardiovascular system during hypoxic development. FASEB J. 2020;34:16504–16515. doi: 10.1096/fj.202001743R 33094855 10.1096/fj.202001743R

[47] Gundersen HJG Bendtsen TF Korbo L Marcussen N Moller A Nielsen K Nyengaard JR Pakkenberg B Sorensen FB Vesterby A. Some new, simple and efficient stereological methods and their use in pathological research and diagnosis. APMIS. 1988;96:379–394. doi: 10.1111/j.1699-0463.1988.tb05320.x 3288247 10.1111/j.1699-0463.1988.tb05320.x

[48] Richter HG Camm EJ Modi BN Naeem F Cross CM Cindrova-Davies T Spasic-Boskovic O Dunster C Mudway IS Kelly FJ. Ascorbate prevents placental oxidative stress and enhances birth weight in hypoxic pregnancy in rats. J Physiol. 2012;590:1377–1387. doi: 10.1113/jphysiol.2011.226340 22289909 10.1113/jphysiol.2011.226340 PMC3382329

[49] Richter HG Hansell JA Raut S Giussani DA. Melatonin improves placental efficiency and birth weight and increases the placental expression of antioxidant enzymes in undernourished pregnancy. J Pineal Res. 2009;46:357–364. doi: 10.1111/j.1600-079x.2009.00671.x 19552758 10.1111/j.1600-079X.2009.00671.x

[50] Romero-Calvo I Ocón B Martínez-Moya P Suárez MD Zarzuelo A Martínez-Augustin O de Medina FS. Reversible Ponceau staining as a loading control alternative to actin in Western blots. Anal Biochem. 2010;401:318–320. doi: 10.1016/j.ab.2010.02.036 20206115 10.1016/j.ab.2010.02.036

[51] Lauer T Kleinbongard P Kelm M. Indexes of NO bioavailability in human blood. Physiology. 2002;17:251–255. doi: 10.1152/nips.01405.2002 10.1152/nips.01405.2002 12433980

[52] Sproston NR Ashworth JJ. Role of C-reactive protein at sites of inflammation and infection. Front Immunol. 2018;9:754. doi: 10.3389/fimmu.2018.00754 29706967 10.3389/fimmu.2018.00754 PMC5908901

[53] Lövgren T Hemmilä I Pettersson K Eskola JU Bertoft E. Determination of hormones by time-resolved fluoroimmunoassay. Talanta. 1984;31:909–916. doi: 10.1016/0039-9140(84)80220-9 18963767 10.1016/0039-9140(84)80220-9

[54] Monie I. Comparative development of the nervous, respiratory, and cardiovascular systems. Environ Health Perspect. 1976;18:55–60. doi: 10.1289/ehp.761855 829490 10.1289/ehp.761855 PMC1475299

[55] Rueda-Clausen CF Morton JS Davidge ST. The early origins of cardiovascular health and disease: who, when, and how. Semin Reprod Med. 2011;29:197–210. doi: 10.1055/s-0031-1275520 21710396 10.1055/s-0031-1275520

[56] Derks JB Giussani DA Jenkins SL Wentworth RA Visser GH Padbury JF Nathanielsz PW. A comparative study of cardiovascular, endocrine and behavioural effects of betamethasone and dexamethasone administration to fetal sheep. J Physiol. 1997;499:217–226. doi: 10.1113/jphysiol.1997.sp021922 9061651 10.1113/jphysiol.1997.sp021922 PMC1159348

[57] Fletcher AJW McGarrigle HHG Edwards CMB Fowden AL Giussani DA. Effects of low dose dexamethasone treatment on basal cardiovascular and endocrine function in fetal sheep during late gestation. J Physiol. 2002;545:649–660. doi: 10.1113/jphysiol.2001.015693 12456840 10.1113/jphysiol.2001.015693 PMC2290705

[58] Garrud TAC Giussani DA. Combined antioxidant and glucocorticoid therapy for safer treatment of preterm birth. Trends Endocrinol Metab. 2019;30:258–269. doi: 10.1016/j.tem.2019.02.003 30850263 10.1016/j.tem.2019.02.003

[59] Jellyman JK Fletcher AJW Fowden AL Giussani DA. Glucocorticoid maturation of fetal cardiovascular function. Trends Mol Med. 2020;26:170–184. doi: 10.1016/j.molmed.2019.09.005 31718939 10.1016/j.molmed.2019.09.005

[60] Fletcher AJ Gardner DS Edwards CMB Fowden AL Giussani DA. Cardiovascular and endocrine responses to acute hypoxaemia during and following dexamethasone infusion in the ovine fetus. J Physiol. 2003;549:271–287. doi: 10.1113/jphysiol.2002.036418 12665612 10.1113/jphysiol.2002.036418 PMC2342926

[61] Doyle LW Ford GW Davis NM Callanan C. Antenatal corticosteroid therapy and blood pressure at 14 years of age in preterm children. Clin Sci (Lond). 2000;98:137–142. 10657267

[62] Kamphuis P de Vries W Bakker J Kavelaars A van Dijk J Schipper M van Oosterhout M Croiset G Heijnen C van Bel F. Reduced life expectancy in rats after neonatal dexamethasone treatment. Pediatr Res. 2007;61:72–76. doi: 10.1203/01.pdr.0000249980.95264.dd 17211144 10.1203/01.pdr.0000249980.95264.dd

[63] Yeh T Lin Y Hsieh W Lin H Lin C Chen J Kao H Chien C. Early postnatal dexamethasone therapy for the prevention of chronic lung disease in preterm infants with respiratory distress syndrome: a multicenter clinical trial. Pediatrics. 1997;100:E3. doi: 10.1542/peds.100.4.e3 10.1542/peds.100.4.e3 9310536

[64] Shaltout HA Rose JC Figueroa JP Chappell MC Diz DI Averill DB. Acute AT1-receptor blockade reverses the hemodynamic and baroreflex impairment in adult sheep exposed to antenatal betamethasone. Am J Physiol Heart Circ Physiol. 2010;299:H541–H547. doi: 10.1152/ajpheart.00100.2010 20543085 10.1152/ajpheart.00100.2010 PMC2930394

[65] Torrens C Kelsall CJ Hopkins LA Anthony FW Curzen NP Hanson MA. Atorvastatin restores endothelial function in offspring of protein-restricted rats in a cholesterol-independent manner. Hypertension. 2009;53:661–667. doi: 10.1161/hypertensionaha.108.122820 19221211 10.1161/HYPERTENSIONAHA.108.122820

[66] Wyrwoll CS Noble J Thomson A Tesic D Miller MR Rog-Zielinska EA Moran CM Seckl JR Chapman KE Holmes MC. Pravastatin ameliorates placental vascular defects, fetal growth, and cardiac function in a model of glucocorticoid excess. Proc Natl Acad Sci USA. 2016;113:6265–6270. doi: 10.1073/pnas.1520356113 27185937 10.1073/pnas.1520356113 PMC4896723

[67] Dec GW Fuster V. Idiopathic dilated cardiomyopathy. N Engl J Med. 1994;331:1564–1575. doi: 10.1056/nejm199412083312307 7969328 10.1056/NEJM199412083312307

[68] Fowden AL Forhead AJ. Glucocorticoids as regulatory signals during intrauterine development. Exp Physiol. 2015;100:1477–1487. doi: 10.1113/ep085212 26040783 10.1113/EP085212

[69] Kimura H Esumi H. Reciprocal regulation between nitric oxide and vascular endothelial growth factor in angiogenesis. Acta Biochim Pol. 2003;50:49–59. doi: 10.18388/abp.2003_3713 12673346

[70] Kuwabara M Kakinuma Y Ando M Katare RG Yamasaki F Doi Y Sato T. Nitric oxide stimulates vascular endothelial growth factor production in cardiomyocytes involved in angiogenesis. J Physiol Sci. 2006;56:95–101. doi: 10.2170/physiolsci.rp002305 16779917 10.2170/physiolsci.rp002305

[71] Teulings N Garrud T Niu Y Skeffington K Beck C Itani N Conlon FG Botting KJ Nicholas LM Ashmore TJ. Isolating adverse effects of glucocorticoids on the embryonic cardiovascular system. FASEB J. 2020;34:9664–9677. doi: 10.1096/fj.202000697R 32502311 10.1096/fj.202000697R PMC7611332

[72] Cianflone E Cappetta D Mancuso T Sabatino J Marino F Scalise M Albanese M Salatino A Parrotta EI Cuda G. Statins stimulate new myocyte formation after myocardial infarction by activating growth and differentiation of the endogenous cardiac stem cells. Int J Mol Sci. 2020;21:7927. doi: 10.3390/ijms21217927 33114544 10.3390/ijms21217927 PMC7663580

[73] Porter KE Turner NA. Statins and myocardial remodelling: cell and molecular pathways. Expert Rev Mol Med. 2011;13:e22. doi: 10.1017/S1462399411001931 21718586 10.1017/S1462399411001931

[74] Bolli R Dawn B. The cornucopia of “pleiotropic” actions of statins: myogenesis as a new mechanism for statin-induced benefits?. Circ Res. 2009;104:144–146. doi: 10.1161/circresaha.108.192500 19179666 10.1161/CIRCRESAHA.108.192500 PMC2746462

[75] Su J Fang M Tian B Luo J Jin C Wang X Ning Z Li X. Atorvastatin protects cardiac progenitor cells from hypoxia-induced cell growth inhibition via MEG3/miR-22/HMGB1 pathway. Acta Biochim Biophys Sin. 2018;50:1257–1265. doi: 10.1093/abbs/gmy133 30481260 10.1093/abbs/gmy133

[76] Longui CA. Glucocorticoid therapy: minimizing side effects. J Pediatr (Rio J). 2007;83:S163–S171. doi: 10.2223/JPED.1713 18000630 10.2223/JPED.1713

[77] Giussani DA Camm EJ Niu Y Richter HG Blanco CE Gottschalk R Blake EZ Horder KA Thakor AS Hansell JA. Developmental programming of cardiovascular dysfunction by prenatal hypoxia and oxidative stress. PLoS One. 2012;7:e31017. doi: 10.1371/journal.pone.0031017 22348036 10.1371/journal.pone.0031017 PMC3278440

[78] Brain KL Allison BJ Niu Y Cross CM Itani N Kane AD Herrera EA Skeffington KL Botting KJ Giussani DA. Intervention against hypertension in the next generation programmed by developmental hypoxia. PLoS Biol. 2019;17:e2006552. doi: 10.1371/journal.pbio.2006552 30668572 10.1371/journal.pbio.2006552 PMC6342530

[79] Massaro M Zampolli A Scoditti E Carluccio MA Storelli C Distante A De Caterina R. Statins inhibit cyclooxygenase-2 and matrix metalloproteinase-9 in human endothelial cells: anti-angiogenic actions possibly contributing to plaque stability. Cardiovasc Res. 2009;86:311–320. doi: 10.1093/cvr/cvp375 19946014 10.1093/cvr/cvp375

[80] Shimokawa H Yasutake H Fujii K Owada MK Nakaike R Fukumoto Y Takayanagi T Nagao T Egashira K Fujishima M. The importance of the hyperpolarizing mechanism increases as the vessel size decreases in endothelium-dependent relaxations in rat mesenteric circulation. J Cardiovasc Pharmacol. 1996;28:703–711. doi: 10.1097/00005344-199611000-00014 8945685 10.1097/00005344-199611000-00014

[81] McGillick EV Orgeig S Allison BJ Brain KL Niu Y Itani N Skeffington KL Kane AD Herrera EA Morrison JL. Molecular regulation of lung maturation in near-term fetal sheep by maternal daily vitamin C treatment in late gestation. Pediatr Res. 2022;91:828–838. doi: 10.1038/s41390-021-01489-4 33859366 10.1038/s41390-021-01489-4 PMC9064793

[82] Sandovici I Fernandez-Twinn DS Hufnagel A Constância M Ozanne SE. Sex differences in the intergenerational inheritance of metabolic traits. Nat Metab. 2022;4:507–523. doi: 10.1038/s42255-022-00570-4 35637347 10.1038/s42255-022-00570-4

