# Supplementary material for: Combined Statin and Glucocorticoid Therapy for the Safer Treatment of Preterm Birth
Source: Hypertension. 2023 Feb 1;80(4):837–51. doi: 10.1161/HYPERTENSIONAHA.122.19647 (PMC10017302; doi:10.1161/HYPERTENSIONAHA.122.19647)
Supplement: Supplementary file 1 [file hyp-80-837-s001.doc]

**Combined statin and glucocorticoid therapy for the safer treatment of preterm birth**

Andrew D. Kane^1^†, Emilio A. Herrera^1^†, Youguo Niu ^1,5,6^, Emily J. Camm ^1^, Beth J. Allison^1^, Deodata Tijsseling^2^, Ciara Lusby^1^, Jan B. Derks ^2^, Kirsty L. Brain^1^, Inge M. Bronckers^4^, Christine M. Cross^1^, Lindsey Berends^3^ & Dino A. Giussani^1,5,6^

†*These authors contributed equally to this study*

^1^ Department of Physiology, Development and Neuroscience, University of Cambridge, Cambridge, UK

^2^ Perinatal Center, University Medical Center, Utrecht, the Netherlands

^3^ University of Cambridge Metabolic Research Laboratories, Institute of Metabolic Science, Addenbrooke's Hospital, Cambridge, UK

^4^ Department of Obstetrics and Gynecology, Radboud University Nijmegen Medical Centre, The Netherlands

^5^ The Cambridge BHF Centre for Research Excellence, Cambridge, UK

^6^ The Cambridge Strategic Research Initiative in Reproduction, Cambridge, UK

**Short Title:**  Statin protection for perinatal glucocorticoids

**Correspondence:** Prof. Dino A. Giussani PhD ScD FRCOG

Department of Physiology Development & Neuroscience

University of Cambridge

Downing Street

Cambridge

CB2 3EG

UK

Tel: +44 1223 333894

E-mail: [dag26@cam.ac.uk](mailto:dag26@cam.ac.uk)


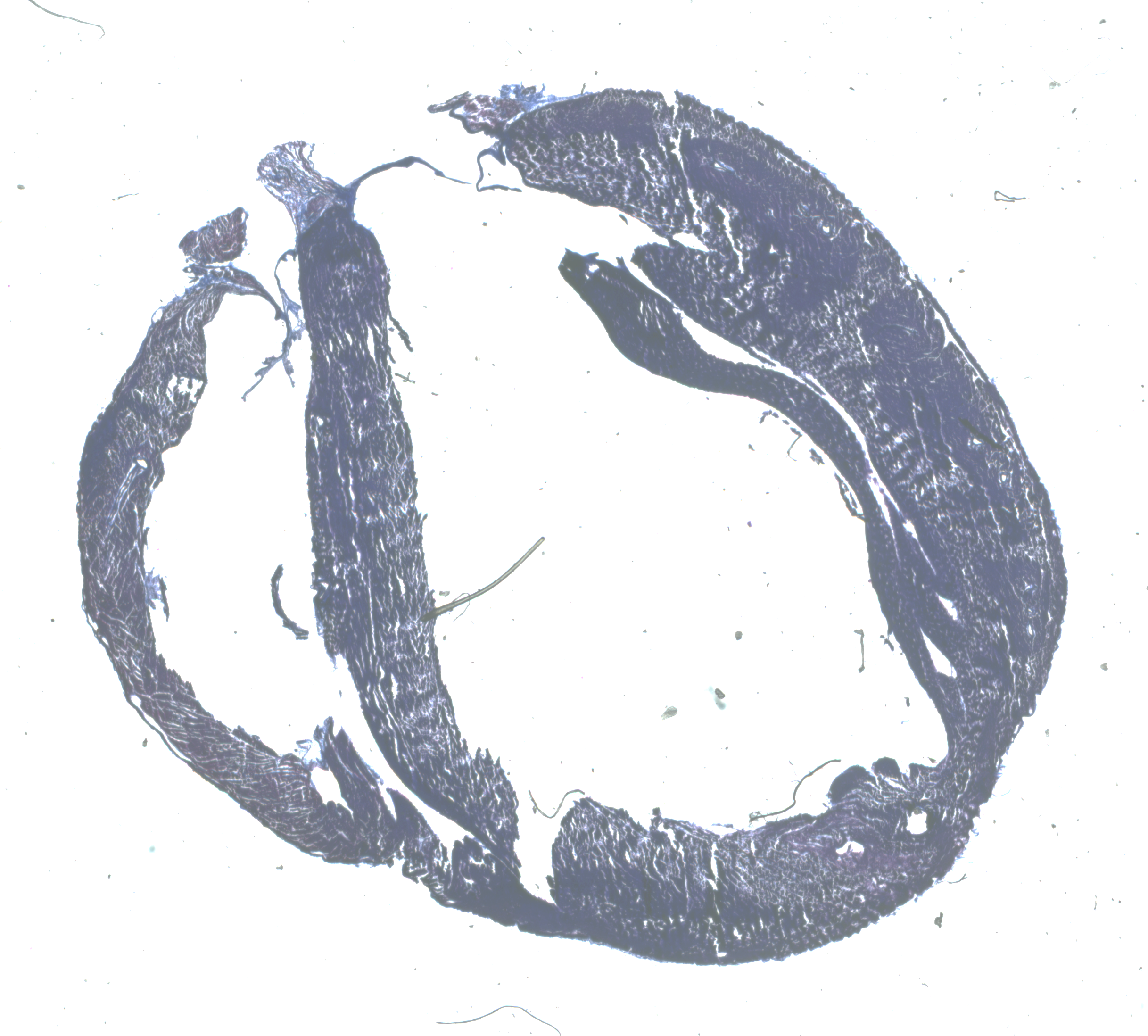

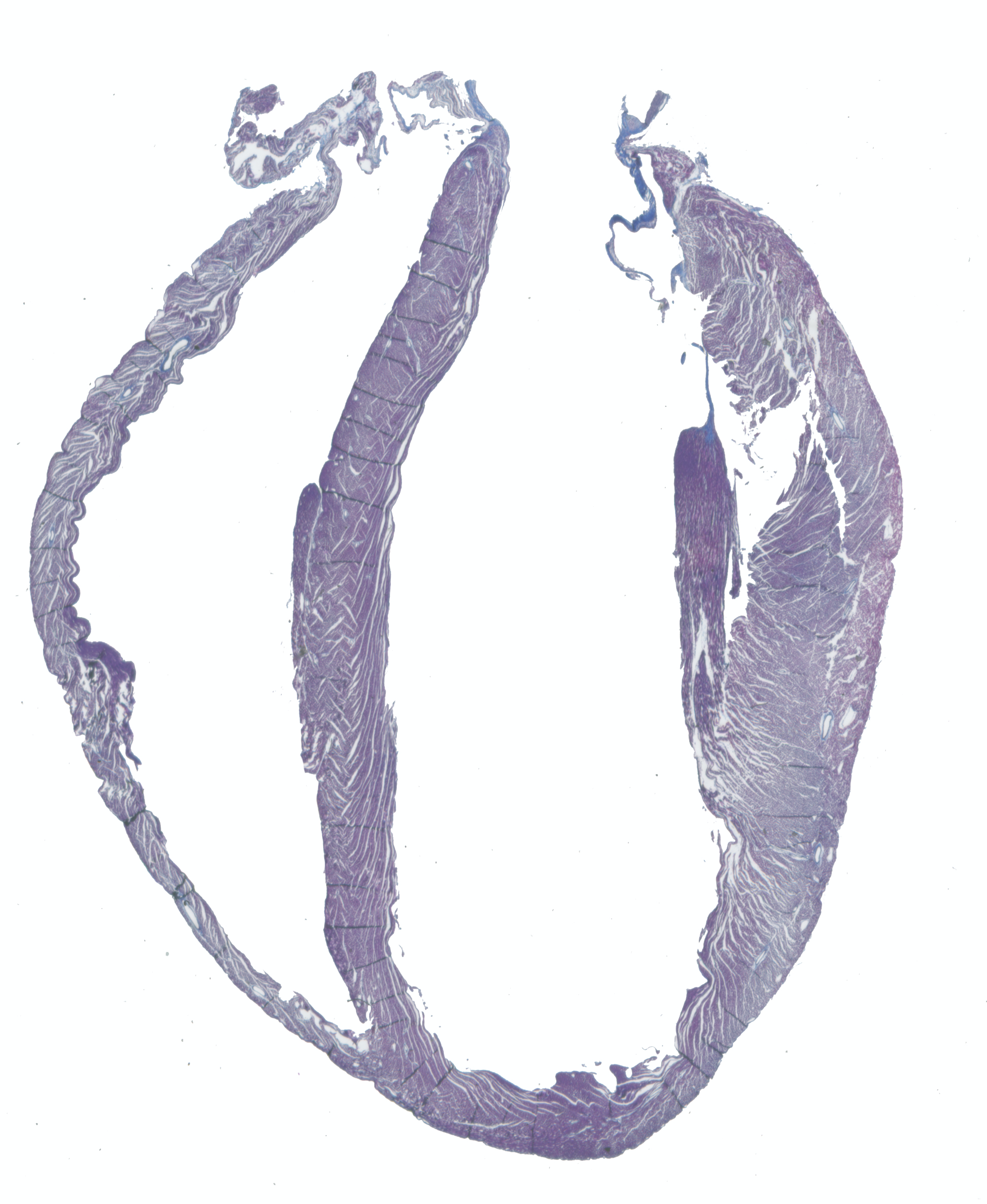

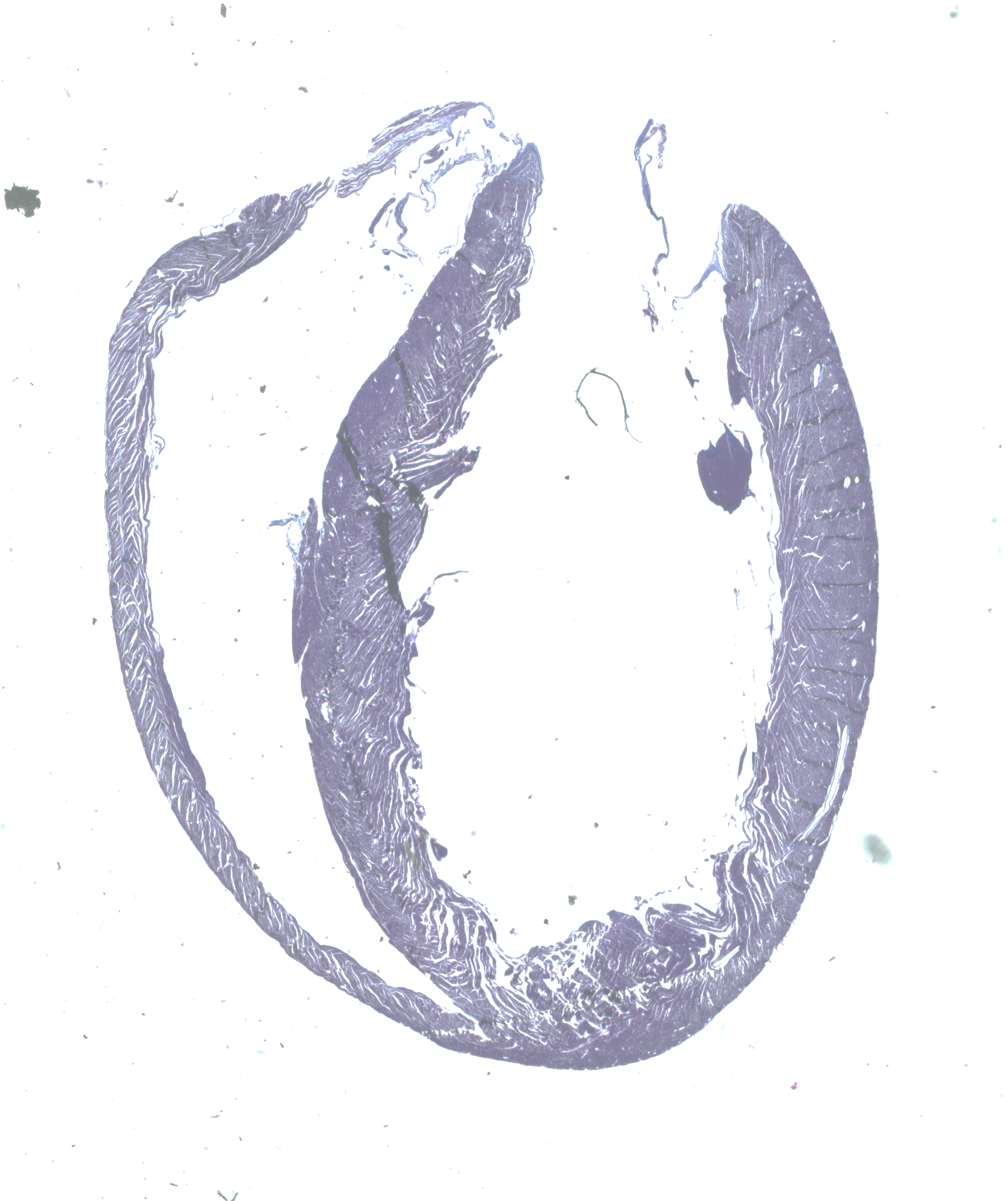

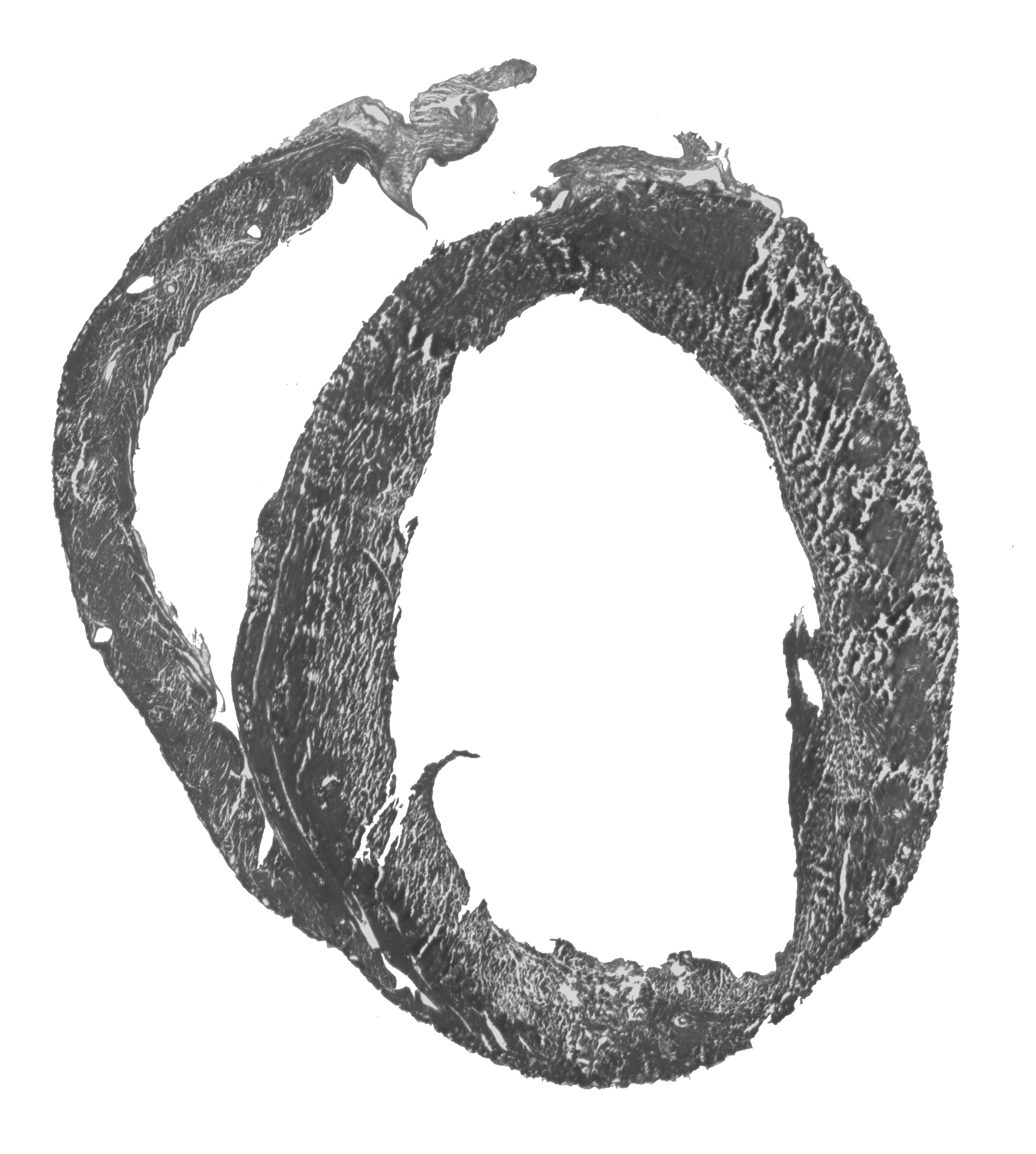


**C**

**D**

**DP**

**P**

LL

RL

RV

LV

IVS

**Figure S1. Cardiac morphology. Examples of mid-cardiac coronal sections of hearts from Control (C), Dex (D), Dex/Pravastatin (DP) and Pravastatin (P) treated animals. Scale bar = 2mm. LV, left ventricle; LL, left lumen; RV, right ventricle; RL, right lumen; IVS, interventricular septum.**
